# Dock2 affects the host susceptibility to *Citrobacter rodentium* infection through regulating gut microbiota

**DOI:** 10.1186/s13099-021-00449-x

**Published:** 2021-08-14

**Authors:** Yuan Xie, Jun Chen, Bing Wu, Tiansheng He, Lu Xie, Zhiping Liu

**Affiliations:** 1 Department of Nephrology, The First People’s Hospital of Nankang District, Ganzhou, Jiangxi China; 2 Gannan Medical University, Ganzhou, Jiangxi China; 3 School of Basic Medicine, Gannan Medical University, Jiangxi Ganzhou, China; 4 Center for Immunology, Key Laboratory of Prevention and Treatment of Cardiovascular and Cerebrovascular Diseases, Ministry of Education, Gannan Medical University, Jiangxi Ganzhou, China

**Keywords:** Gut microbiota, Dock2, *Citrobacter rodentium*, Colitis, 16S rRNA gene sequencing

## Abstract

**Background:**

Dysregulated gut microbiota is one of major pathogenic factors in the development of colitis. Dock2 acts as a guanine nucleotide exchange factor (GEF) and activates small G protein RAC1. Our previous study showed that, compared to wild type (WT) mice, *Dock2*^*−/−*^ mice were more susceptible to colitis induced by *Citrobacter rodentium* infection. However, it is not clear whether gut microbiota affects the host susceptibility to enteric bacterial infection in *Dock2*^*−/−*^ mice.

**Results:**

In this study, we demonstrated that Dock2 regulated the gut microbiota and affected the host susceptibility to *C. rodentium* infection by co-housing, fecal microbiota transfer and antibiotic treatment methods. Microbiota analysis by 16 S rRNA gene sequencing showed that Dock2 increased the abundance of *prevotellaceae-NK3B31-group* and *Lactobacillus* but decreased that of *Helicobacter*.

**Conclusions:**

These results suggest that Dock2 regulates the composition of gut microbiota and affects the host susceptibility to *C. rodentium* infection.

**Supplementary Information:**

The online version contains supplementary material available at 10.1186/s13099-021-00449-x.

## Introduction

Inflammatory bowel disease (IBD), including Crohn’s disease (CD) and ulcerative colitis (UC), causes chronic relapsing inflammatory disorders in genetically susceptible individuals [1, 2]. The pathogenesis of IBD involves dysregulation of gut microbiota, genetic susceptibility and immune abnormalities. Gut microbiota plays a crucial role in regulating host responses to pathogens and maintaining intestinal homeostasis. Dysregulated gut microbiota may suppress mucosal immune system, damage intestinal epithelial cells, increase the intestinal permeability, thus inducing intestinal inflammation [3].

However, the exact pathogenesis of IBD is still unclear. IBD animal model is an important means to study the pathogenesis. Among them, *Citrobacter rodentium* infection is often used to study the formation of colitis [4]. *C. rodentium* is a gram-negative bacterium in the intestine, which can induce intestinal proliferation and inflammation. Different from another commonly used colitis model which is induced by innate immune response by the treatment of dextran sodium sulfate, *C. rodentium* infection induces host immune response involving with both innate and adaptive immunity, which is more similar to IBD patients [4].

Dedicator of cytokinesis 2 (Dock2) is a guanine exchange factor (GEFs), which specifically activates the small G protein RAC1 by mediating GTP-GDP exchange and regulates the formation of cytoskeleton [5]. Our previous study showed that *Dock2*^*−/−*^ mice were more susceptible to colitis induced by *C. rodentium* infection, which was mainly manifested by higher mortality, weight loss, *C. rodentium* load and intestinal damage [6]. However, the precise mechanism by which Dock2 regulates the host susceptibility to *C. rodentium* infection remains largely unclear.

Numerous studies suggested that microbiota composition was critical for host resistance to *C. rodentium* infection [7]. Compared to conventional mice, germ-free mice could not clear the enteric pathogen at late stage of *C. rodentium* infection [8]. The commensal *Escherichia coli*, but not *Bacteroides* species, could outcompete luminal *C. rodentium* by utilizing metabolites such as monosaccharides [8]. The probiotic *Lactobacillus*, either used alone or with other probiotics, could inhibit the intestinal inflammation induced by *C. rodentium* infection through inducing the production of IL-22 and activating regulatory T cells [9]. *Segmented filamentous bacteria (SFB)*, an anaerobic commensal bacterium tightly adherent to intestinal epithelial cells, enhanced the host resistance to *C. rodentium* infection by inducing intestinal Th17 cells [10, 11]. The abundance of *Lachnospiraceae*, one of major short-chain fatty acid-producing commensal bacteria, was correlated with less intestinal inflammation induced by *C. rodentium* infection [12]. Administration of *Clostridiales* conferred the host protection to *C. rodentium* infection in neonatal mice that lack *Clostridiales* while depletion of *Clostridiales* abolished colonization resistance in adult mice, indicating that *Clostridiales* enhanced host resistance to *C. rodentium* infection [13]. Under fiber-deprived diet, mucus-degrading commensals, such as *Akkermansia muciniphila*, could shift from metabolizing dietary polysaccharides to degrading mucus glycan, thus damaging mucus barrier and increasing host susceptibility to *C. rodentium* infection [14]. However, the roles of Dock2 in the regulation of gut microbiota and host susceptibility to *C. rodentium* infection are unknown.

Here, we tested the impact of Dock2 on the composition of gut microbiota and the host susceptibility to *C. rodentium* infection by co-housing, gut microbiota transfer and antibiotic treatment experiments. Moreover, we detected the specific gut microbiota changes by 16 S rRNA sequencing. This study elucidated the effects of gut microbiota on host susceptibility of *Dock2*^−/−^ mice to *C. rodentium* infection and provided a new theoretical basis for the treatment of intestinal inflammatory diseases.

## Materials and methods

### Mice

Wild-type (WT) and *Dock2*^*−/−*^ mice on C57BL/6 background were provided by Yoshinori Fukui (Kyushu University, Japan). Mice were housed in SPF environment of Experimental Animal Center of Gannan Medical University. The lights were adjusted to simulate normal day and night, and mice had ad libitum access to sterile drinking water. The 4 to 6 week-old mice were selected. Except that the mice selected for the cohousing experiment were female mice to prevent fighting against each other, the other mice were either female or male mice(Same gender in the same cage). This study was approved by the Ethics Committee of Gannan Medical University.

### Co-housing experiment

The cohousing experiment methods were performed as previously described with modification [15]. Specifically, female mice were used for cohousing experiment to prevent the fighting against each other. WT and *Dock2*^*−/−*^ mice were housed in the same cages for 4 weeks. After that, the mice were separated into different cages according to their genotypes and then infected with *C. rodentium*. Fecal samples of mice before and after co-house were collected and stored at − 80 ℃.

### Gut microbiota transfer experiment

As previously described [16], Each mouse was fed with streptomycin (20 mg dissolved in 100 µl PBS solution). Then all mice were divided into two groups. One group received the gut microbiota from WT mice, and the other received the gut microbiota from *Dock2*^*−/−*^ mice from the previous cohort. WT and *Dock2*^*−/−*^ mice (n = 3–4 for each genotype) were selected as donors. Fresh feces were taken from 9 to 10 AM every day (To ensure that the feces of the two type donors were of the similar quality each time), and the fecal transplantation were completed within 1 h after the feces were taken. Specifically, the same weights of feces were homogenized in 0.05 % cystine HCl PBS and each mouse received 100 µl supernatant every other day. After six consecutive times, mice were infected with *C. rodentium*. Fecal samples were collected before and after streptomycin treatment and after 6 times of gut microbiota transfer.

### Antibiotic treatment experiment

As previously described [17], antibiotic cocktail (1 mg/ml ampicilln, 1 mg/ml metronidazole, 1 mg/ml neomycin, and 0.5 mg/ml vancomycin) was added into the drinking water for 4 weeks. One day after the termination of antibiotic treatment, the mice were infected with *C. rodentium*.

### *Citobacter rodentium *infection

*C. rodentium* was cultured overnight and then sub-cultured into longitude phase. The mice were orally administrated with *C. rodentium* at the dose of 1 × 10^10^ CFU/mouse as previously described [6]. To determine the bacterial load, feces were homogenized and serially diluted in PBS, then the diluent was plated onto McConkey agar plates for CFU counting. After infection, mice were sacrificed. Colons were collected and colon lengths were measured.

### 16 S rRNA gene sequencing and data processing of gut microbiota

Genomic DNA from feces was extracted using fecal DNA extraction kit. The construction of high-throughput sequencing library and sequencing based on Illumina miseq platform were completed by GENEWIZ (Suzhou, China). A series of PCR primers were used to amplify two highly variable regions (V3 and V4) of prokaryotic 16 S rDNA. The upstream primers contained the sequence “CCTACGGRRBGCASCAKVGAAT” and the downstream primers contained the sequence “GGACTACNVGGGTWTCTAATCC”. In addition, the end of 16 S rDNA PCR product was inserted with an “Index” connector by PCR to facilitate NGS sequencing. The quality of the library was detected by Agilent 2100 Biological Analyzer (Agilent Technologies, Palo Alto, CA, USA), and the library concentration was detected by Qubit 2.0 Fluorometer (Invitrogen, Carlsbad, CA). After the DNA library was mixed, PE250/300 double-ended sequencing was performed according to the instruction manual of Illumina miseq (Illumina, San Diego, CA, USA), and the sequence information was read by Miseq Control Software (MCS) of Miseq.

Analysis of 16 S rRNA gene sequencing data generated in this study was performed according to the sequencing data processing steps as described in [18].

The differences in bacteria among various groups were analyzed by a variety of analysis methods, including heatmaps, Anosim, Metastasis, and LEfSe analysis.

### Analysis of inter group differences in α diversity index

This is based on the α diversity index table, using R language to construct the box chart, which can show the maximum and minimum values, median and abnormal values of α diversity index of each group, and also can directly reflect the diversity degree between groups. It mainly includes Chao1 analysis and Shannon analysis. This analysis uses the Chao1 algorithm to estimate the number of OTUs in the samples, which is often used to evaluate the total number of species in ecology. Shannon analysis was used to estimate microbial diversity in samples.

### Anosim analysis (analysis of similarities)

Similarity analysis is a nonparametric test, which is used to test whether the difference between groups (two or more groups) is significantly greater than that within the group to judge whether the grouping is meaningful. The input data for the ANOSIM test were from the normalized OTU tables. The main reference values are R value and P value. R: R value range [− 1, 1], the actual result is generally [0, 1]. R value close to 0 means that there is no significant difference between groups and within groups, and R value close to 1 means that the difference between groups is greater than the difference within groups.

### Sibling mice experiment

Our mouse breeding cages were *Dock2*^*+/−*^ mice and *Dock2*^*+/−*^ mice. The offspring from these breeding cages contained *Dock2*^*+/+*^ (WT) mice and *Dock2*^*−/−*^ mice. In this way, these two genotype mice had the same parents and were reared in the same environments starting from their birth. These mice were fed in the same cages until the age of 4 weeks old and fecal samples were collected. After that, mice were separated according to their genotype. At the age of 10 weeks old, fecal samples of mice were collected again.

### Histology

The colon tissue was fixed in 10 % formalin, embedded in paraffin, sectioned (5 μm) and stained with hematoxylin and eosin (H & E). The sections were scored by the pathologist blindly based on the degree of inflammation, edema, hyperplasia, colon injury, and crypt length as previously described [19]. According to the progressive score, the degree of lesion was mainly determined by the depth of infiltration, the number of inflammatory cells and the degree of crypt damage. The score of inflammation range and degree: normal = 0; mild = 1; moderate = 2; severe = 3; the score of inflammation infiltration: normal = 0; mucosa = 1; submucosa = 2; full layer = 3; crypt damage degree: normal = 0; one third of basement crypt was destroyed = 1; two thirds of basement crypt was destroyed = 2; only complete surface epithelium = 3; all crypt and epithelium were destroyed = 4. The highest score is 10.

### Statistical analysis

GraphPad prism 8.0 was used for data analysis. The data was represented by mean ± standard error (SEM). *P* value was calculated by ANOVA or Student t test. *P < 0.05 was considered as significantly different. The results of 16 S rRNA sequencing were analyzed by nonparametric statistical analysis. Identification of significant taxa between groups was performed using both Metastats [20] and LEfSe [21] software.

## Results

### Dock2 regulates the gut microbiota and affects the host susceptibility to ***Citrobacter rodentium*** infection

**Fig. 1:**
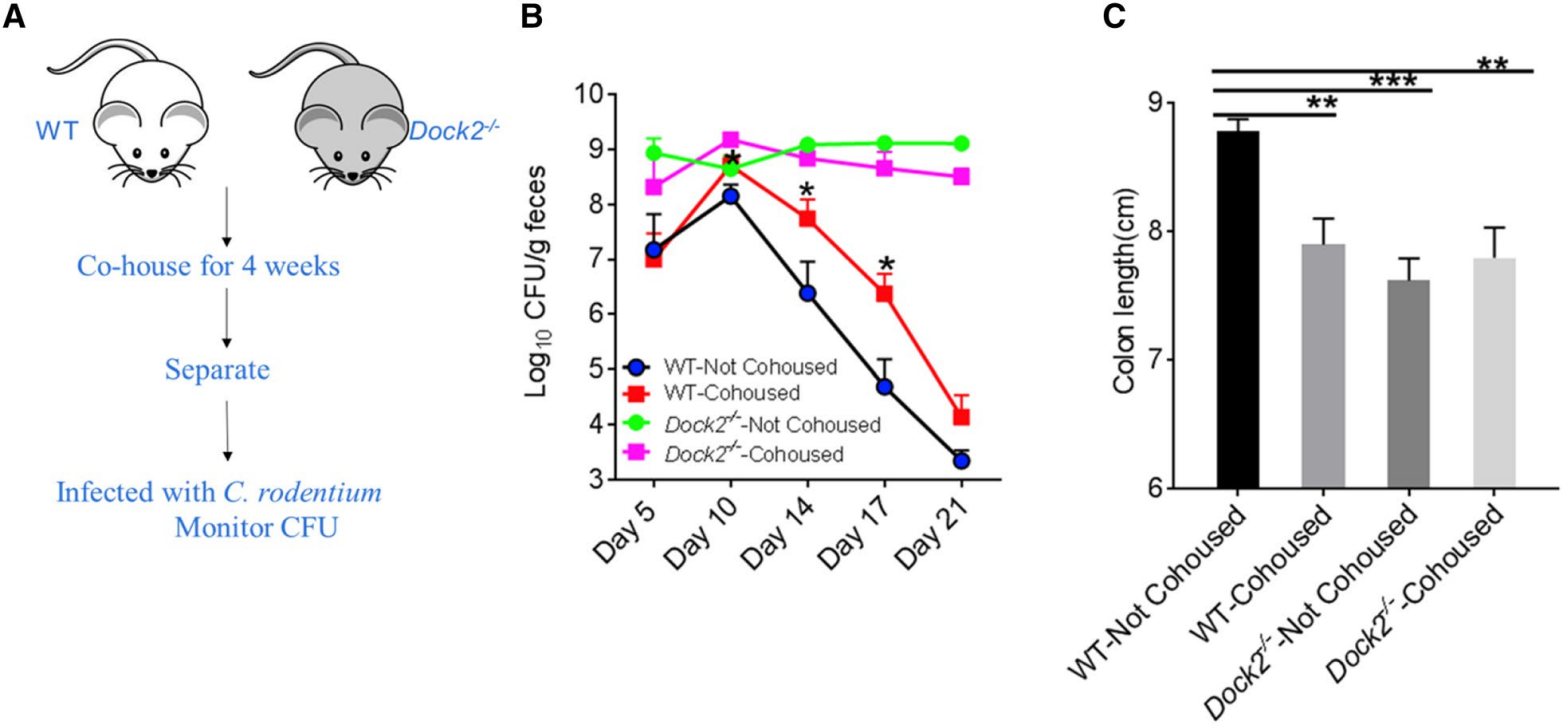
WT mice cohoused with *Dock2*^−/−^ mice have a higher bacterial load and shorter colonic length after *C. rodentium* infection than WT mice without cohousing. **A** Schematic diagram of Cohousing experiments. WT and *Dock2*^*−/−*^ mice were not cohoused or cohoused for 4 weeks and infected with *C. rodentium.* **B** The amount of *C. rodentium* in the feces on days 5, 10, 14, 17, and 21 after infection. **C** Colon length on day 21 after infection. WT-cohoused, n = 15; *Dock2*^*−/−*^-cohoused, n = 9; WT not cohoused, n = 10; *Dock2*^*−/−*^-not cohoused, n = 4. * *p* < 0.05, ** *p* < 0.01, *** *p* < 0.001 vs. Not cohoused WT. Results were representative of two independent experiments

Female WT mice were cohoused or not cohoused with *Dock2*^*−/−*^mice for 4 weeks and then infected with *C. rodentium* (Fig. 1A). Five days after infection, there was no difference in *C. rodentium* load of WT mice with or without cohousing. However, WT mice cohoused with *Dock2*^*−/−*^ mice had significantly more *C. rodentium* load than those not cohoused at 10, 14, and 17 days after infection (Fig. 1B). Consistent with this result, the cohoused WT mice had shorter colon lengths than those not cohoused at 21 days after infection (Fig. 1C). In contrast, no difference in *C. rodentium* load was identified in *Dock2*^*−/−*^ mice with or without cohousing throughout the experiment. These results indicated that WT and *Dock2*^*−/−*^ mice had differences in composition of gut microbiota, and WT mice might obtain gut microbiota from *Dock2*^*−/−*^ mice by co-housing, which increased their susceptibility to *C. rodentium* infection.

**Fig. 2:**
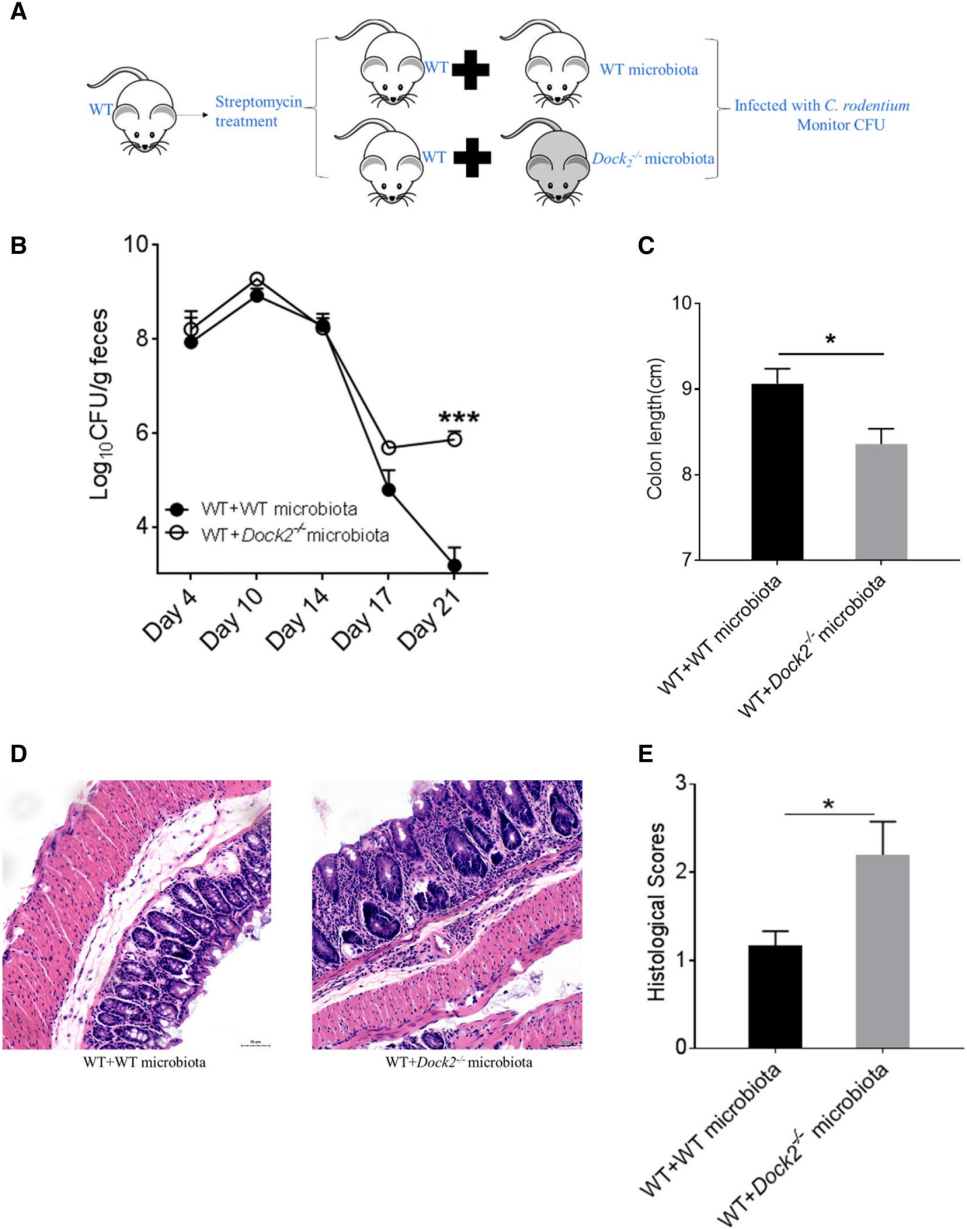
WT mice transferred with gut microbiota of *Dock2*^−/−^ mice have more bacterial load, shorter colon length, and more severe intestinal inflammation than WT mice transferred with WT mouse gut microbiota. **A** Schematic diagram of gut microbiota transfer experiments. WT mice were treated with streptomycin and transferred with WT or *Dock2*^*−/−*^ mouse gut microbiota, then infected with *C. rodentium*. Mouse feces were collected to measure bacterial load on days 4, 10, 14, 17, and 21. Mice were sacrificed on day 21 and colon lengths were measured. **B** *C. rodentium* bacterial load. **C** Colon length on day 21. **D**, **E** H & E staining photograph of colonic tissues. Scale bar 50 μm. WT mice n = 6; *Dock2*^*−/−*^ mice n = 5. * *p* < 0.05, *** *p* < 0.001. Data were presented as mean ± SEM. Results were representative of two independent experiments

In order to further test whether the difference in gut microbiota between WT and *Dock2*^*−/−*^mice affects the host susceptibility to *C. rodentium* infection, we used a direct gut microbiota transfer experiment as previously described (Fig. 2A) [16]. On days 4, 7, 10, and 14 after *C. rodentium* infection, there was no difference in the *C. rodentium* load of WT mice between transferred with WT mouse microbiota and with *Dock2*^*−/−*^ mouse microbiota, indicating that *Dock2*^*−/−*^ mouse gut microbiota had no significant effect on *C. rodentium* load in early stage of infection. However, on day 21 after infection, the *C. rodentium* load of WT mice transferred with *Dock2*^*−/−*^ mouse microbiota was significantly higher (Fig. 2B). Consistently, the colon lengths of WT transferred with *Dock2*^*−/−*^ mouse microbiota were significantly shorter than those transferred with WT mouse microbiota controls (Fig. 2C). Furthermore, H & E staining results showed that the colonic tissues of WT mice transferred with *Dock2*^*−/−*^ mouse microbiota group had more serious crypt hyperplasia and more inflammatory cell infiltration than those in controls (Fig. 2D, E). The results collectively supported that WT and *Dock2*^*−/−*^ mice had difference in gut microbiota, which might affect the host susceptibility to *C. rodentium* infection.

**Fig. 3:**
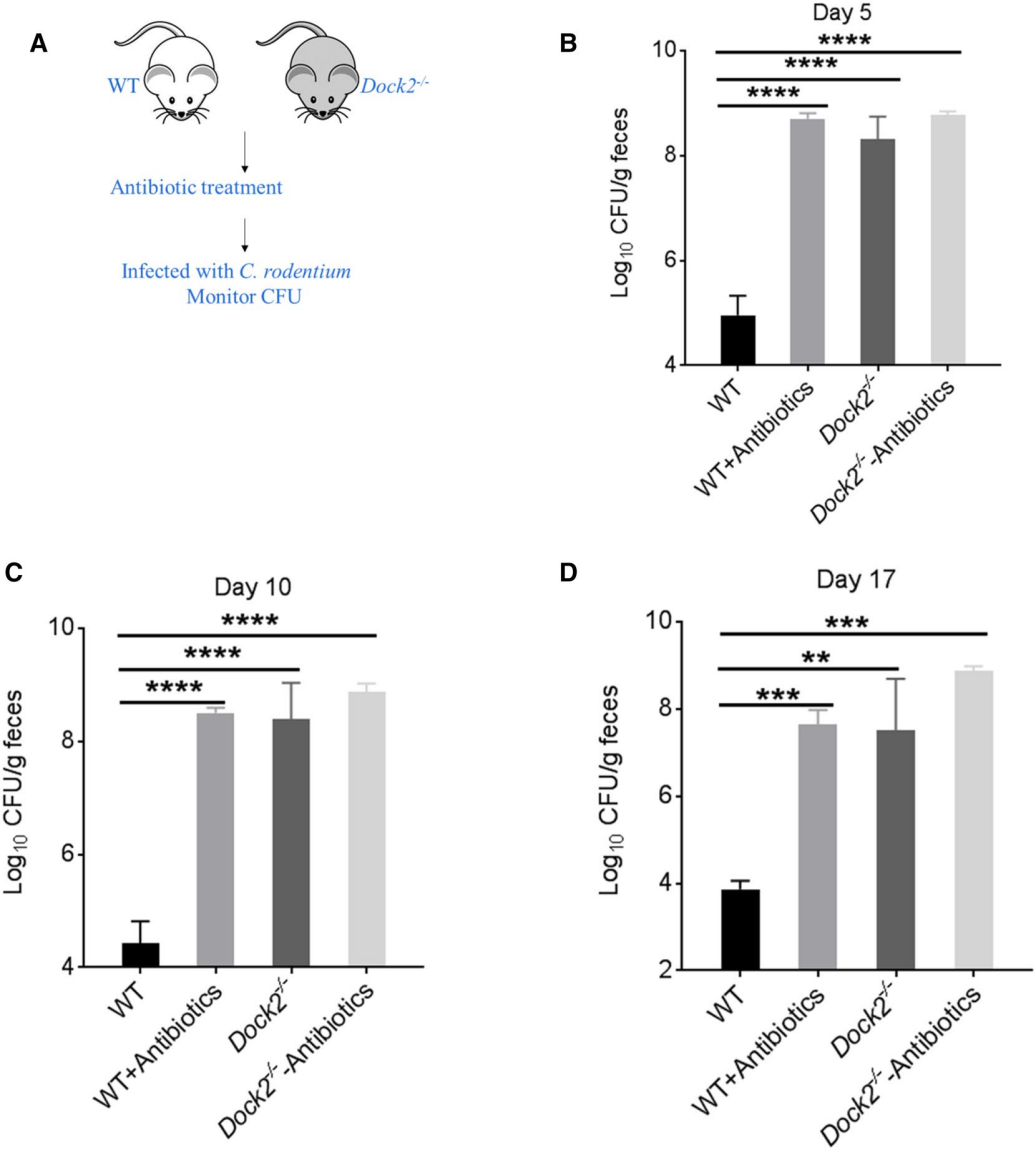
Antibiotic treatment reversed the difference in the host susceptibility of WT and *Dock2*^−/−^ mice to *C. rodentium* infection. **A** Schematic diagram of antibiotic treatment experiments. WT and *Dock2*^*−/−*^mice were infected with *C. rodentium* after 4 weeks of antibiotic treatment. Feces were collected on days 5 (**B**), 10 (**C**), and 17 (**D**) for *C. rodentium* load testing. At day 5, WT, n = 7; WT + Antibiotics, n = 4; *Dock2*^*−/*−^, n = 6; *Dock2*^*−/−*^+Antibiotics, n = 5. At day10, WT, n = 5; WT + Antibiotics, n = 4; *Dock2*^*−/−*,^ n = 5; *Dock2*^*−/−*^+Antibiotics, n = 4. At day17, WT n = 5; WT + Antibiotics n = 4; *Dock2*^*−/−*^ n = 3; *Dock2*^*−/−*^+Antibiotics n = 3. ** *p* < 0.01, *** *p* < 0.001, **** *p* < 0.0001 vs. WT. Data were presented as mean ± SEM. Results were representative of two independent experiments

To further certify the results, antibiotic treatment experiment was performed (Fig. 3A). Our results showed that both WT and *Dock2*^*−/−*^ mice treated with antibiotic could not clear *C. rodentium* on days 5, 10 and 17 day after infection, which indicated that removal of gut microbiota reversed the difference of bacterial loads (Fig. 3B–D). These results suggest that the gut microbiota was critical for the host to eliminate *C. rodentium* infection, and the host susceptibility to *C. rodentium* infection was closely associated with the composition of gut microbiota.

The difference of gut microbiota between sibling WT and *Dock2*^−/−^ mice.

**Fig. 4:**
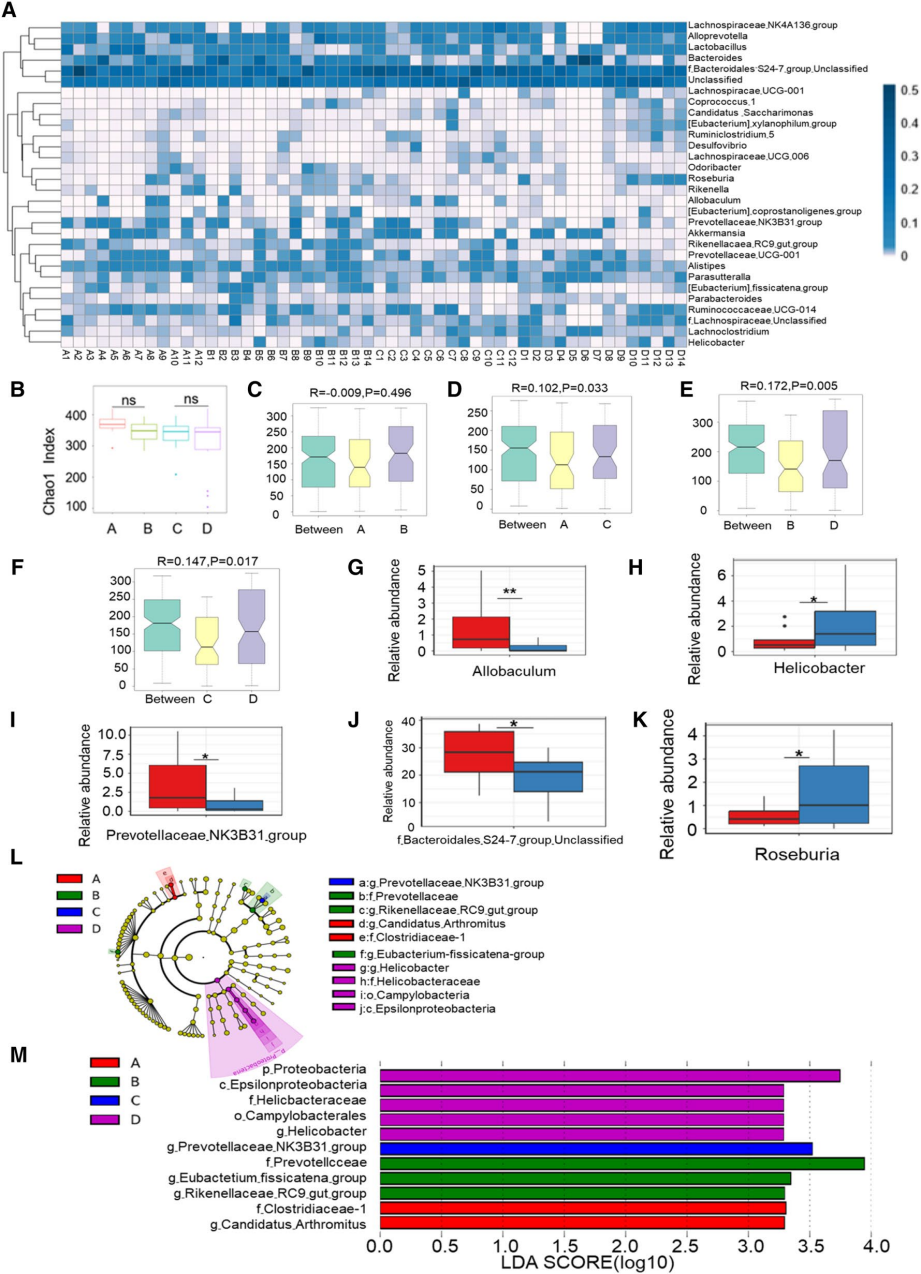
16 S rRNA gene sequencing results of gut microbiota of siblings of WT and *Dock2*^−/−^ mice. Feces were collected from WT and *Dock2*^*−/−*^ mice before and after cage division (4 weeks and 10 weeks). DNA was extracted, and submitted to16S rRNA gene sequencing analysis. **A** The heat map of the gene level based on the 30 most abundant OTUs in the sequencing results; ** B** The microbiota abundance index Chao1 diversity analysis; **C**–**F** Anosim test results; **G**–**K** Metastats test results; **L**, **M** LEfSe test results; **A** represented the siblings of WT mice before cage division, **B** represented the siblings of *Dock2*^*−/*−^mice before cage division, **C** represented siblings of WT mice at 6 weeks after cage division, and **D** represented the siblings of *Dock2*^*−/−*^ mice at 6 weeks after cage division. * *p* < 0.05, ** *p* < 0.01, ns, not significantly different

We selected sibling WT and *Dock2*^*−/−*^ mice, produced by mating *Dock2*^*+/−*^ and *Dock2*^*+/−*^ mice. The gut microbiota of sibling WT and *Dock2*^*−/ −*^mice were analyzed by 16 S rRNA gene sequencing. Heat map results showed that *Dock2*^*−/−*^ mice possessed more abundance in *Helicobacter*, *Roseburia* and *Lachnoclostridium*, while less abundance in *Prevotellaceae-NK3B31-group* compared to WT mice (Fig. 4A).

Our alpha diversity analysis based on Chao1 and Shannon indices showed that there is no significant difference in the species richness and diversity of gut microbiota between the sibling WT and Dock2-/- mice when examined at 4 weeks old of age and 10 weeks old of age (6 weeks after cage division), respectively (Fig. 4B and Additional file 1: Figure S1). Anosim test results showed no significant difference in the composition of gut microbiota between WT and *Dock2*^*−/−*^ mice at 4 weeks of age (Fig. 4C). This could be explained by the fact that WT mice and *Dock2*^*−/−*^ mice were cohoused before 4 weeks of age. Anosim test results for WT mice between 4 weeks and 10 weeks of age were R = 0.102 and p = 0.033, indicating that there is a significant but slight gut microbial composition difference in the WT mice before and after cage division (Fig. 4D). Anosim test results for *Dock2*^*−/−*^ mice between 4 and 10 weeks of age were R = 0.172 and *p* = 0.005, suggesting that the gut microbiota of *Dock2*^*−/−*^ mice before and after cage division was also significantly different (Fig. 4E). Moreover, the composition of gut microbiota in WT and *Dock2*^*−/−*^ mice at 10 weeks old of age was significantly different according to Anosim test (Fig. 4F). The results showed that the gut microbiota became different after cage division possibly due to different genotypes.

We next analyzed the gut microbiota difference between sibling WT and *Dock2*^*−/−*^ mice after cage division. Metastats test results showed that *Dock2*^*−/−*^ mice had higher contents of *Roseburia* and *Helicobacter*, lower contents of *Allobaculum, Bacteroidales-S24-7-group-unclassified*, and *Prevotellaceae -NK3B31- group* (Fig. 4G–K). The LEfSe test chart showed that the gut microbiota difference between WT and *Dock2*^*−/−*^ mice was *prevotellaceae-NK3B31-group, proteobacteria*, Epsilonproteobacteria, Helicobacteraceae, *Campylobacterales, and Helicobacter* (Fig. 4L, M). In short, at 6 weeks after cage division, *Dock2*^*−/−*^ mice had decreased *Prevotellaceae-NK3B31-group* and increased *Helicobacter* in the gut microbiota compared to WT mice.

### The difference of gut microbiota before and after co-housing

**Fig. 5:**
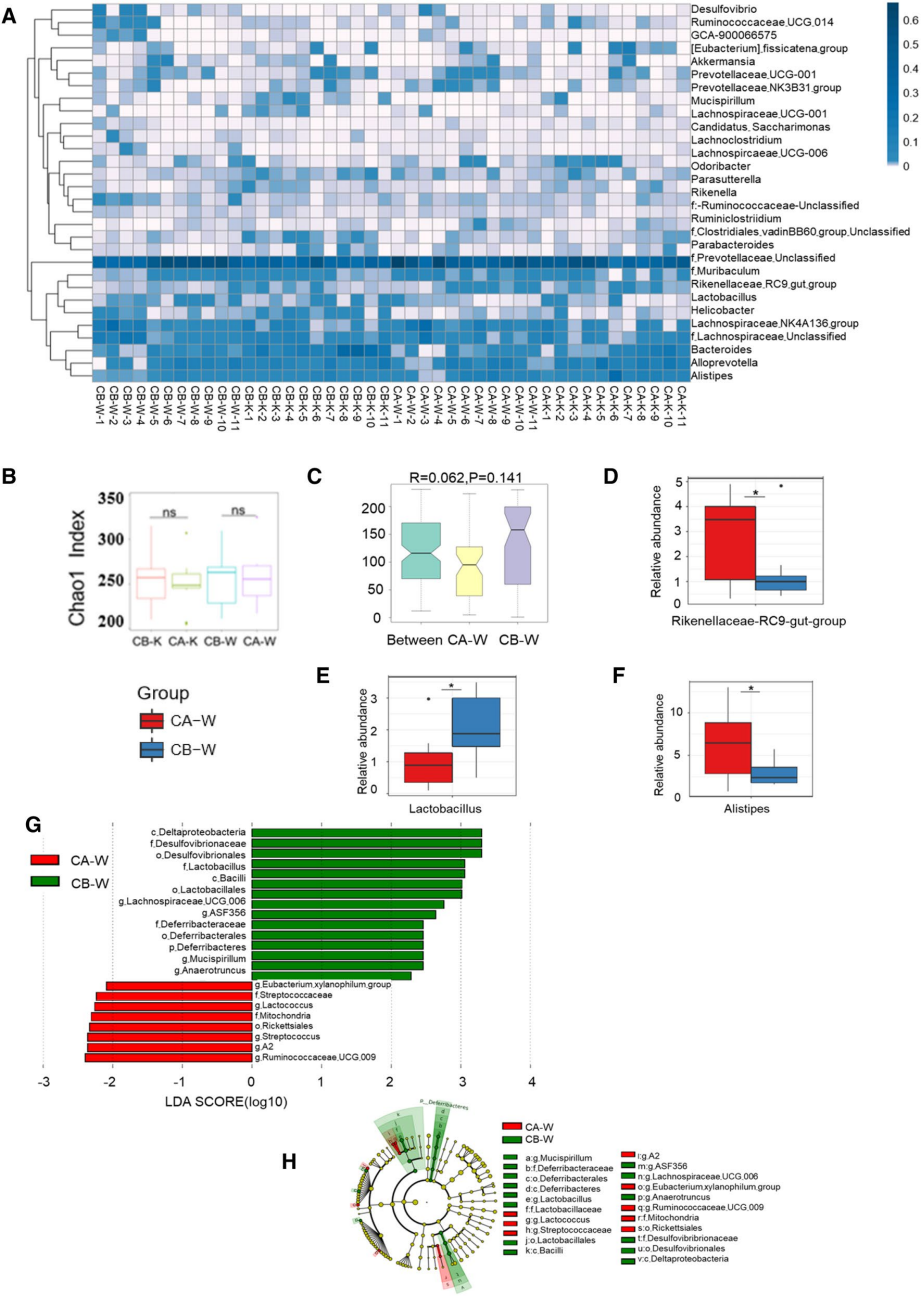
16 S rRNA gene Sequencing results of WT and *Dock2*^***−/−***^mouse gut microbiota before and after cohousing. Feces were collected from WT and *Dock2*^*−/−*^mice before and after cohousing, and submitted to DNA extraction for 16 S rRNA gene sequencing analysis. **A** Genus heat map based on the 30 most abundant OTUs in sequencing results; **B** The microbiota abundance index Chao1diversity analysis; **C** Anosim test results; **D**–**F** Metastats test results; **G**, **H** LEfSe test results; CB-W represented WT mice before cohousing, CB-K represented *Dock2*^*−/−*^mice before cohousing, CA-W represented WT mice 4 weeks after cohousing, and CA-K represented *Dock2*^*−/−*^mice after cohousing. * *p* < 0.05, ** *p* < 0.01, ns, not significantly different

As described in Fig. 1, WT mice co-housed with *Dock2*^*−/−*^ mice got certain gut microbiota from *Dock2*^*−/−*^ mice and reduced the resistance to *C. rodentium* infection. To detect the specific exchanged gut microbiota, the analysis of 16 S rRNA gene sequencing was performed. The results showed that WT mice cohoused with *Dock2*^*−/−*^ mice had increased *Rikenellaceae-RC9-gut-group* and reduced *Lactobacillus* and *Desulfovibrio* (Fig. 5A). Alpha diversity analysis demonstrated that there was no significant difference in the richness and diversity of gut microbiota in both the WT and *Dock2*^*−/−*^ mice when they were each compared before and after the co-housing experiment (Fig. 5B and Additional file 1: Figure S2). The Anosim test, on the other hand, showed no significance difference in the gut microbial profiles of WT mice before and after co-housing (Fig. 5C). However, Metastats test results showed that WT mice cohoused with *Dock2*^*−/−*^ mice had higher load of *Alisipes* and *Rikenellaceae-RC9-gut-group* and lower load of *Lactobacillus* (Fig. 5D–F).

LEfSe test results showed that co-housing reduced the relative abundance of Deltaproteobacteria, Dessulfovibrionaceae, Dessulfovibrionales, *Lachnospiraceae-UCG006, ASF356*, Lactobacillaceae, *Lactobacillus Bacilli*, Deferribacteraceae, Deferribacterales, *Defferribacteres, Mucispirillum*, and *Anaerotruncus*, while reduced the relative abundance of *Eubacterium-xylanophilum-group, Streptococcaceae, Rickettsiales, Lactococcus, Streptococcus, Mitochondria, A2* and *Runinococcaceae-UCG-009* (Fig. 5G, H). Based on those analysis, we inferred that WT mice cohoused with *Dock2*^*−/−*^ mice possessed lower abundance of *Lactobacillus*, which might be associated with increased host susceptibility to *C. rodentium* infection.

### Gut microbiota analysis in transfer experiment

**Fig. 6:**
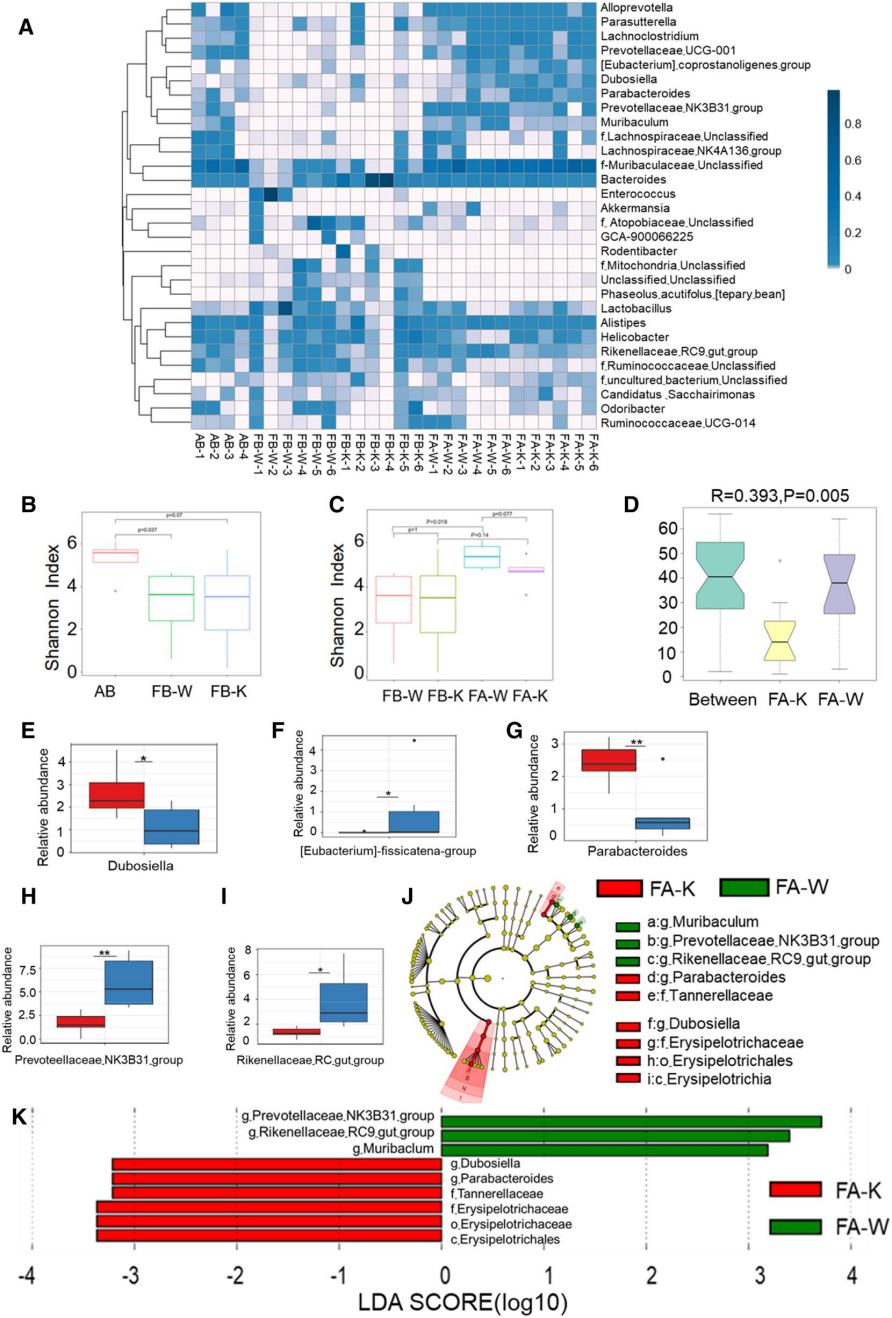
16 S rRNA Sequencing results of WT and *Dock2*^***−/−***^ gut microbiota before and after transfer to WT mice. WT mice were treated with streptomycin and then transferred with gut microbiota of WT or *Dock2*^*−/−*^. Fecal samples were collected before and after gut microbiota transfer. DNA were extracted for 16 S rRNA gene sequencing analysis. **A** The heat map of the 30 most abundant OTUs in the sequencing results. **B**, **C** The microbiota abundance index shannon diversity analysis; **D** Anosim test result results. **E**–**I** Metastats test results. **J**, **K** LEfSe test results. AB represented mice before streptomycin treatment; FB-W represented WT mice before transfer, after which they were transferred with WT gut microbiota; FB-K represented for WT mice before transfer, after which they were transferred with *Dock2*^*−/−*^ mouse gut microbiota. FA-W represented WT mice, to which the WT mouse microbiota was transferred; FA-K represented WT mice, to which the *Dock2*^*−/−*^ mouse microbiota was transferred. * *p* < 0.05, ** *p* < 0.01, ns, not significantly different

In order to determine specific gut microbiota relating to the host susceptibility to *C. rodentium* infection, we performed 16 S rRNA gene sequencing after the gut microbiota transfer experiment as shown in Fig. 2. The results showed that WT mice receiving *Dock2*^*−/−*^ mouse microbiota (FA-K) had higher levels of bacteria including *Helicobacter, Dubosiella, Parabacteroides*, and lower levels of bacteria including *Prevotellaceae-NK3B31-group, g-Muribaculum- Unclassified, Lachnospiraceae- NK4A136-group, Lachnospiraceae -Unclassified* (Fig. 6A). Based on the alpha diversity analysis, it was shown that the gut bacterial species diversity, not species richness, was significantly affected in the mice involved in the fecal transplant experiment. After antibiotic treatment and prior to the fecal transplant procedure, both FB-W and FB-K mice had a significant (p = 0.037) and a marginally significant (p = 0.07) reduction in microbial species diversity, respectively, when compared to that of the treatment naïve WT mice based on Shannon anlaysis but not Chao1 analysis(Fig. 6B and Additional file 1: Figure S3). After fecal transplant, a significant restoration of microbial species diversity was observed only in mice receiving the WT fecal material (FA-W group) (p = 0.019), but not in those which had the *Dock2*^*−/−*^ fecal material (FA-K group) (Fig. 6C). The Anosim test result further demonstrated that the gut microbiota of the FA-K and FA-W groups are significantly different from each other (R = 0.393, p = 0.005), as depicted in Fig. 6D.

The Metastats test showed that WT mice receiving *Dock2*^*−/−*^ mice gut microbiota had lower levels of bacteria including *Prevotellaceae-NK3B31-group, Rikenellaceae-RC9-gut-group [Eubacterium]-fissicatena-group* and higher levels of bacteria including *Dubosiella* and *Parabacteroides* (Fig. 6E–I). The relative abundance between groups was further analyzed by LEfSe test, which showed that WT mice (FA-W) possessed *Prevotellaceae-NK3B31-group; Rikenellaceae-RC9- gut-group; Muribaculum*; while *Dock2*^*−/−*^ bacteria (FA-K) mice possessed *Dubosiella; Parabacteroides;* Tannerellaceae; Erysipelotrichaceae; Erysipelotrichales; Erysipelotrichia (Fig. 6J, K). Those analysis showed that WT mice transferred with *Dock2*^*−/−*^ mouse gut microbiota had fewer *Prevotellaceae-NK3B31-group* than the WT mice transferred with WT gut microbiota, which was consistent with the results of the gut microbiota in the siblings experiment.

In summary, *Dock2*^*−/−*^ mice have fewer *Prevotellaceae-NK3B31-group, Lactobacillus* and higher *Helicobacter* than the WT mice. *Prevotellaceae-NK3B31 -group* and *Lactobacillus* may be beneficial for the host to defend against *C. rodentium* infection, while *Helicobacter* may aggravate the host susceptibility to *C. rodentium* infection.

## Discussion

Our previous study showed that *Dock2*^*−/−*^ mice were more susceptible to colitis induced by *C. rodentium* infection than WT mice [6]. However, the exact mechanism by which Dock2 protects host from enteric bacterial infection or colitis has not been described. In this study, we demonstrated that Dock2 plays a key role in the response to *C. rodentium* infection through regulating gut microbiota by co-housing, fecal microbiota transfer and antibiotic treatment methods. Furthermore, using 16 S rRNA gene sequencing analysis, we showed that Dock2 induced the increase in the abundance of *prevotellaceae-NK3B31-group* and *Lactobacillus* and the decrease in the abundance of *Helicobacter*.

The gut microbiota is a key player in mammalian physiology and participates in the protection against *C. rodentium* infection. Studies have shown that gene deficiency could alter the composition of gut microbiota and affect the host susceptibility to intestinal pathogens. For instance, it has been reported that alteration in the composition of gut microbiota caused by Nod2 deficiency gave rise to a reversible risk of colitis in mice, while reciprocal microbiota transplantation reduced disease risk [22]. A study showed that Nlrp6 deficiency in mouse colonic epithelial cells resulted in altered fecal microbiota characterized by expanded representation of bacterial *phyla Bacteroidetes* (*Prevotellaceae*) and *TM7* [23]. Another study showed significant changes in the abundance of the *Firmicutes* and *Bacteroidetes phyla*, when comparing Caspase-1, -7 and − 3 knockout mice to WT mice [24]. Furthermore, the microbiota of Card 9 deficiency mice contributed to the decreased host resistance to *C. rodentium* infection. These lines of evidence implied that the composition of the gut microbiota could be regulated by various genes [24]. Consistent with these findings, we showed that *Dock2* deficiency has significant consequences on the composition of gut microbiota characterized by the decrease in *Prevotellaceae-NK3B31-group* and *Lactobacillus* and the increase in *Helicobacter*, which was linked to the enhanced vulnerability to *C. rodentium* infection.

*Lactobacillus* represents a source of lactic acid-producing probiotic bacteria. Reported benefits of lactobacilli include their ability to activate the host immune system, prevent the duration and intensity of diarrheal episodes, enhance colonization resistance, and produce bacteriocins (pathogen inhibitory compounds) [25]. It was found that expansion of gut *Lactobacilli* communities inhibited the amplification of γ- *Proteobacteria* and actinomycetes, improved the mucosal barrier function, reduced the production of infectious colonic crypt hyperplasia and tissue inflammatory factors, thus enhancing the resistance of mice to *C. rodentium* infection [26]. Numerous studies demonstrated that the *Lactobacilli*-enriched commensal gut microenvironment protects against *C. rodentium* infection and colitis [27]. Therefore, the significant decrease of *Lactobacillus* in the *Dock2*^*−/−*^ mice could mean that they benefit less from the positive effects of *Lactobacillus*, which may render the host more susceptible to *C. rodentium* infection. *Helicobacter pylori* is a gram-negative pathogenic bacterium and its infection in humans has been a challenge due to a higher incidence rate of the disease [28, 29]. *H. pylori* infection is implicated in the pathogenesis of gastritis, gastric ulcer, gastric cancer, gastric mucosa‑associated lymphoid tissue (MALT) lymphoma, IBD, and CRC [30]. The analysis of the gut microbiota in IBD patients showed the increase in Proteobacteria phylum [31]. Studies have shown that *Helicobacte*r genus is prone to induce colitis in gene-deficient mouse animal models [32]. Therefore, the significant increase of *Helicobacter* in the *Dock2*^*−/−*^ mice suggested that these mice were more susceptible to *C. rodentium* than the WT mice.

There is an evidence suggesting that *Prevotella* is associated with opportunistic infections, such as periodontitis or bacterial vaginosis [33]. *Prevotella* has been implicated in rheumatoid arthritis [34], human immunodeficiency virus (HIV) infection [35], and IBD [34]. Expansion of intestinal *Prevotella copri* correlates with enhanced susceptibility to DSS-induced colitis [34]. *Prevotella* promote experimental colitis in mice, indicating that *prevotella* may be harmful.

However, *Prevotella* is also considered to be a common symbiotic bacterium due to its presence in healthy humans, including the mouth, gastrointestinal tract, urogenital tract, and skin. It was reported that the protection of mice from lethal colitis was associated with higher levels of bacteria from *Bacteroidetes* [36]. Prevotellaceae is a branch of *Bacteroidates*, which can promote the production of butyrate, a short-chain fatty acid (SCFA) [37]. SCFAs can enhance the expression of tight junction protein, maintain the integrity of epithelial barrier, and reduce the expression of proinflammatory cytokines in mucus [38]. Stachyose has been reported to improve the intestinal homeostasis in high-fat diet -fed mice by improving the bacterial diversity and the increases in the relative abundances of gut microbiota including *prevotellaceae-NK3B31-group* [39]. In addition, *Prevotella* induced the homeostasis of glucose by regulating gut gluconeogenesis [40]. These findings suggest the beneficial effects of *Prevotella*. In agreement with these studies, our results showed a significant decrease of *Prevotellaceae-NK3B31-group* in the *Dock2*^*−/−*^ mice, which indicated that these mice may have less SCFAs in their intestine and benefit less from the positive effects of *Prevotella. Prevotella* is a large genus with species diversity. Different species may exert different function. In addition, the role of *Prevotella* may depend on the experimental models and disease. Our present finding showed that the decrease of *prevotellaceae-NK3B31-group* in the *Dock2*^−/−^ mice correlates with enhanced susceptibility to *C. rodentium* infection.

## Conclusions

To date, we described a clear link between Dock2 deficiency and gut microbiota composition. Our results indicated that Dock2 deficiency should be added to the list of host genetic factors that may drive alterations in the gut microbiota, which in turn may promote intestinal disease. However, additional studies are needed in future work. For instance, the transfer of *prevotellaceae-NK3B31* to *Dock2*^−/−^ mice is required to verify its biological significance in the host defense against *C. rodentium* infection. Mechanistic details underlying how Dock2 regulates gut microbiota against *C. rodentium* infection also need to be further characterized.

## Supplementary Information

**Additional file 1:Figure S1.** Shannon analysis of 16S rRNA gene sequencing results from gut microbiota of siblings of WT and Dock2^−/−^ mice. Shannon index in alpha diversity analysis of microbial community was shown. A represented the siblings of WT mice before cage division, B represented the siblings of Dock2^−/−^ mice before cage division, C represented siblings of WT mice at 6 weeks after cage division, and D represented the siblings of Dock2^−/−^ mice at 6 weeks after cage division. **Figure S2.** Shannon analysis of 16S rRNA gene Sequencing results from WT and Dock2^−/−^ mouse gut microbiota before and after cohousing. Shannon index in alpha diversity analysis of microbial community was shown. CB-W represented WT mice before cohousing, CB-K represented Dock2^−/−^ mice before cohousing, CA-W represented WT mice at 4 weeks after cohousing, and CA-K represented Dock2^−/−^ mice after cohousing. **Figure S3.** chao1 analysis of 16S rRNA Sequencing results from WT and Dock2^−/−^ gut microbiota before and after transfer to WT mice. Chao1 index in alpha diversity analysis of microbial community was shown. AB represented mice before streptomycin treatment; FB-W represented WT mice before transfer, after which they were transferred with WT gut microbiota; FB-K represented for WT mice before transfer, after which they were transferred with Dock2^−/−^ mouse gut microbiota. FA-W represented WT mice which received WT mouse microbiota; FA-K represented WT mice which received Dock2^−/−^ mouse microbiota.

## Data Availability

The datasets used and/or analysed during the current study are available from the corresponding author on reasonable request.

## References

[1] Torres J (2017). Crohn’s disease. Lancet.

[2] Ungaro R (2017). Ulcerative colitis. Lancet.

[3] Thaiss CA (2016). The microbiome and innate immunity. Nature.

[4] Eckmann L (2006). Animal models of inflammatory bowel disease: lessons from enteric infections. Ann NY Acad Sci.

[5] Namekata K (2014). Dock GEFs and their therapeutic potential: neuroprotection and axon regeneration. Prog Retin Eye Res.

[6] Liu Z (2016). DOCK2 confers immunity and intestinal colonization resistance to Citrobacter rodentium infection. Sci Rep.

[7] Mullineaux-Sanders C (2019). Citrobacter rodentium-host-microbiota interactions: immunity, bioenergetics and metabolism. Nat Rev Microbiol.

[8] Kamada N (2012). Regulated virulence controls the ability of a pathogen to compete with the gut microbiota. Science.

[9] Hrdý J (2020). Lactobacillus reuteri 5454 and Bifidobacterium animalis ssp. lactis 5764 improve colitis while differentially impacting dendritic cells maturation and antimicrobial responses. Sci Rep.

[10] Ivanov II (2009). Induction of intestinal Th17 cells by segmented filamentous bacteria. Cell.

[11] Atarashi K (2015). Th17 cell induction by adhesion of microbes to intestinal epithelial cells. Cell.

[12] Ryu SH (2016). The probiotic lactobacillus prevents Citrobacter rodentium-induced murine colitis in a TLR2-dependent manner. J Microbiol Biotechnol.

[13] Geiger TL (2014). Nfil3 is crucial for development of innate lymphoid cells and host protection against intestinal pathogens. J Exp Med.

[14] Desai MS (2016). A dietary fiber-deprived gut microbiota degrades the colonic mucus barrier and enhances pathogen susceptibility. Cell.

[15] Man SM (2015). Critical role for the DNA sensor AIM2 in stem cell proliferation and cancer. Cell.

[16] Willing BP (2011). Altering host resistance to infections through microbial transplantation. PLoS One.

[17] Kuhn KA (2018). Bacteroidales recruit IL-6-producing intraepithelial lymphocytes in the colon to promote barrier integrity. Mucosal Immunol.

[18] Han Z (2018). Vertical variation of a black soil’s properties in response to freeze-thaw cycles and its links to shift of microbial community structure. Sci Total Environ.

[19] Zaki MH (2011). The NOD-like receptor NLRP12 attenuates colon inflammation and tumorigenesis. Cancer Cell.

[20] White JR, Nagarajan N, Pop M (2009). Statistical methods for detecting differentially abundant features in clinical metagenomic samples. PLoS Comput Biol.

[21] Segata N (2011). Metagenomic biomarker discovery and explanation. Genome Biol.

[22] Couturier-Maillard A (2013). NOD2-mediated dysbiosis predisposes mice to transmissible colitis and colorectal cancer. J Clin Invest.

[23] Elinav E (2011). NLRP6 inflammasome regulates colonic microbial ecology and risk for colitis. Cell.

[24] Brinkman BM (2011). Caspase deficiency alters the murine gut microbiome. Cell Death Dis.

[25] Kumar A (2016). Lactobacillus acidophilus counteracts inhibition of NHE3 and DRA expression and alleviates diarrheal phenotype in mice infected with Citrobacter rodentium. Am J Physiol Gastrointest Liver Physiol.

[26] Jiminez JA (2017). Butyrate supplementation at high concentrations alters enteric bacterial communities and reduces intestinal inflammation in mice infected with Citrobacter rodentium. mSphere.

[27] Jiang Y (2016). Immunological mechanisms involved in probiotic-mediated protection against Citrobacter rodentium-induced colitis. Benef Microbes.

[28] Bohr UR (2004). Identification of enterohepatic Helicobacter species in patients suffering from inflammatory bowel disease. J Clin Microbiol.

[29] Oliveira AG (2006). Isolation of Helicobacter pylori from the intestinal mucosa of patients with Crohn’s disease. Helicobacter.

[30] Inoue I (2014). Helicobacter pylori-related chronic gastritis as a risk factor for colonic neoplasms. World J Gastroenterol.

[31] Gophna U (2006). Differences between tissue-associated intestinal microfloras of patients with Crohn’s disease and ulcerative colitis. J Clin Microbiol.

[32] Zhang L (2005). Natural colonization with Helicobacter species and the development of inflammatory bowel disease in interleukin-10-deficient mice. Helicobacter.

[33] Larsen JM (2017). The immune response to Prevotella bacteria in chronic inflammatory disease. Immunology.

[34] Scher JU (2013). Expansion of intestinal Prevotella copri correlates with enhanced susceptibility to arthritis. Elife.

[35] Dillon SM (2016). Gut dendritic cell activation links an altered colonic microbiome to mucosal and systemic T-cell activation in untreated HIV-1 infection. Mucosal Immunol.

[36] Yue SJ (2019). Berberine treatment-emergent mild diarrhea associated with gut microbiota dysbiosis. Biomed Pharmacother.

[37] Esquivel-Elizondo S (2017). Insights into Butyrate Production in a Controlled Fermentation System via Gene Predictions. mSystems.

[38] Ewaschuk JB (2008). Secreted bioactive factors from Bifidobacterium infantis enhance epithelial cell barrier function. Am J Physiol Gastrointest Liver Physiol.

[39] Liu Y (2019). Regulatory effects of stachyose on colonic and hepatic inflammation, gut microbiota dysbiosis, and peripheral CD4(+) T cell distribution abnormality in high-fat diet-fed mice. J Agric Food Chem.

[40] De Vadder F (2016). Microbiota-Produced Succinate Improves Glucose Homeostasis via Intestinal Gluconeogenesis. Cell Metab.

