# Supplementary material for: Dock2 affects the host susceptibility to Citrobacter rodentium infection through regulating gut microbiota
Source: Gut Pathog. 2021 Aug 14;13:52. doi: 10.1186/s13099-021-00449-x (PMC8364044; doi:10.1186/s13099-021-00449-x)
Supplement: Supplementary file 1 — Additional file 1:Figure S1. Shannon analysis of 16S rRNA gene sequencing results from gut microbiota of siblings of WT and Dock2−/− mice. Shannon index in alpha diversity analysis of microbial community was shown. A represented the siblings of WT mice before cage division, B represented the siblings of Dock2−/− mice before cage division, C represented siblings of WT mice at 6 weeks after cage division, and D represented the siblings of Dock2−/− mice at 6 weeks after cage division. Figure S2. Shannon analysis of 16S rRNA gene Sequencing results from WT and Dock2−/− mouse gut microbiota before and after cohousing. Shannon index in alpha diversity analysis of microbial community was shown. CB-W represented WT mice before cohousing, CB-K represented Dock2−/− mice before cohousing, CA-W represented WT mice at 4 weeks after cohousing, and CA-K represented Dock2−/− mice after cohousing. Figure S3. chao1 analysis of 16S rRNA Sequencing results from WT and Dock2−/− gut microbiota before and after transfer to WT mice. Chao1 index in alpha diversity analysis of microbial community was shown. AB represented mice before streptomycin treatment; FB-W represented WT mice before transfer, after which they were transferred with WT gut microbiota; FB-K represented for WT mice before transfer, after which they were transferred with Dock2−/− mouse gut microbiota. FA-W represented WT mice which received WT mouse microbiota; FA-K represented WT mice which received Dock2−/− mouse microbiota. [file 13099_2021_449_MOESM1_ESM.ppt]

## Slide 1
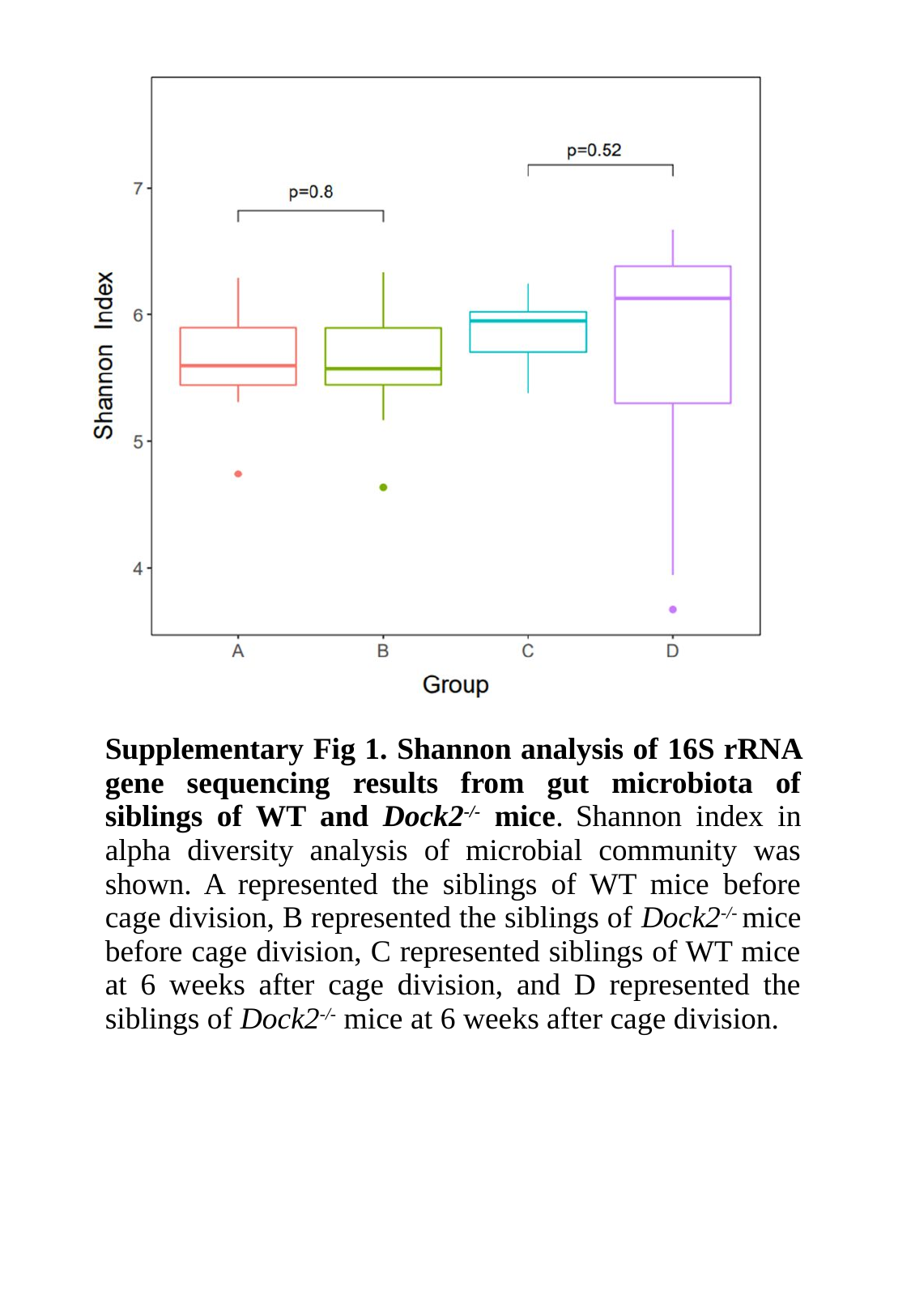

Supplementary Fig 1. Shannon analysis of 16S rRNA gene sequencing results from gut microbiota of siblings of WT and Dock2-/- mice. Shannon index in alpha diversity analysis of microbial community was shown. A represented the siblings of WT mice before cage division, B represented the siblings of Dock2-/- mice before cage division, C represented siblings of WT mice at 6 weeks after cage division, and D represented the siblings of Dock2-/- mice at 6 weeks after cage division.

## Slide 2
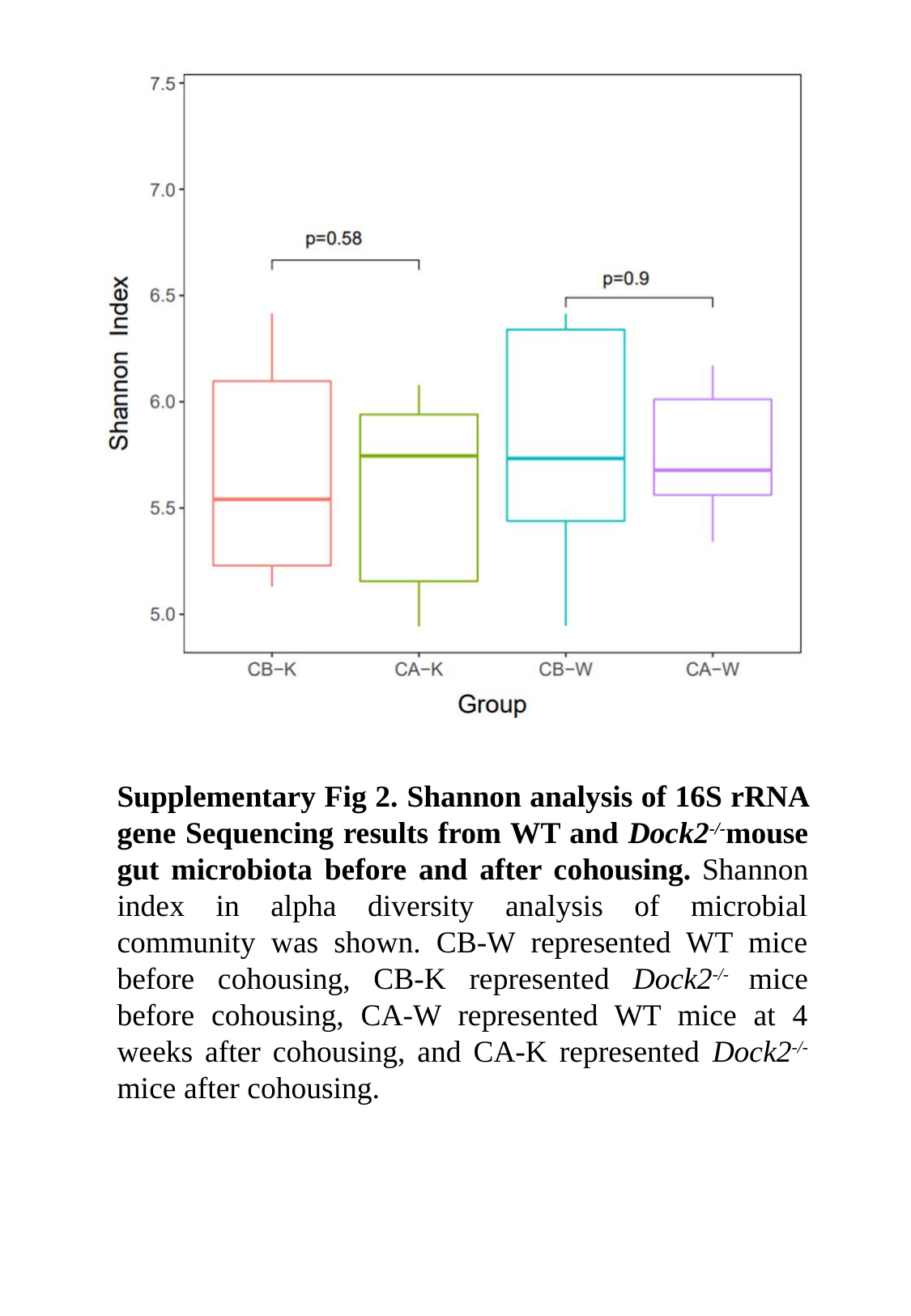

Supplementary Fig 2. Shannon analysis of 16S rRNA gene Sequencing results from WT and Dock2-/-mouse gut microbiota before and after cohousing. Shannon index in alpha diversity analysis of microbial community was shown. CB-W represented WT mice before cohousing, CB-K represented Dock2-/- mice before cohousing, CA-W represented WT mice at 4 weeks after cohousing, and CA-K represented Dock2-/- mice after cohousing.

## Slide 3
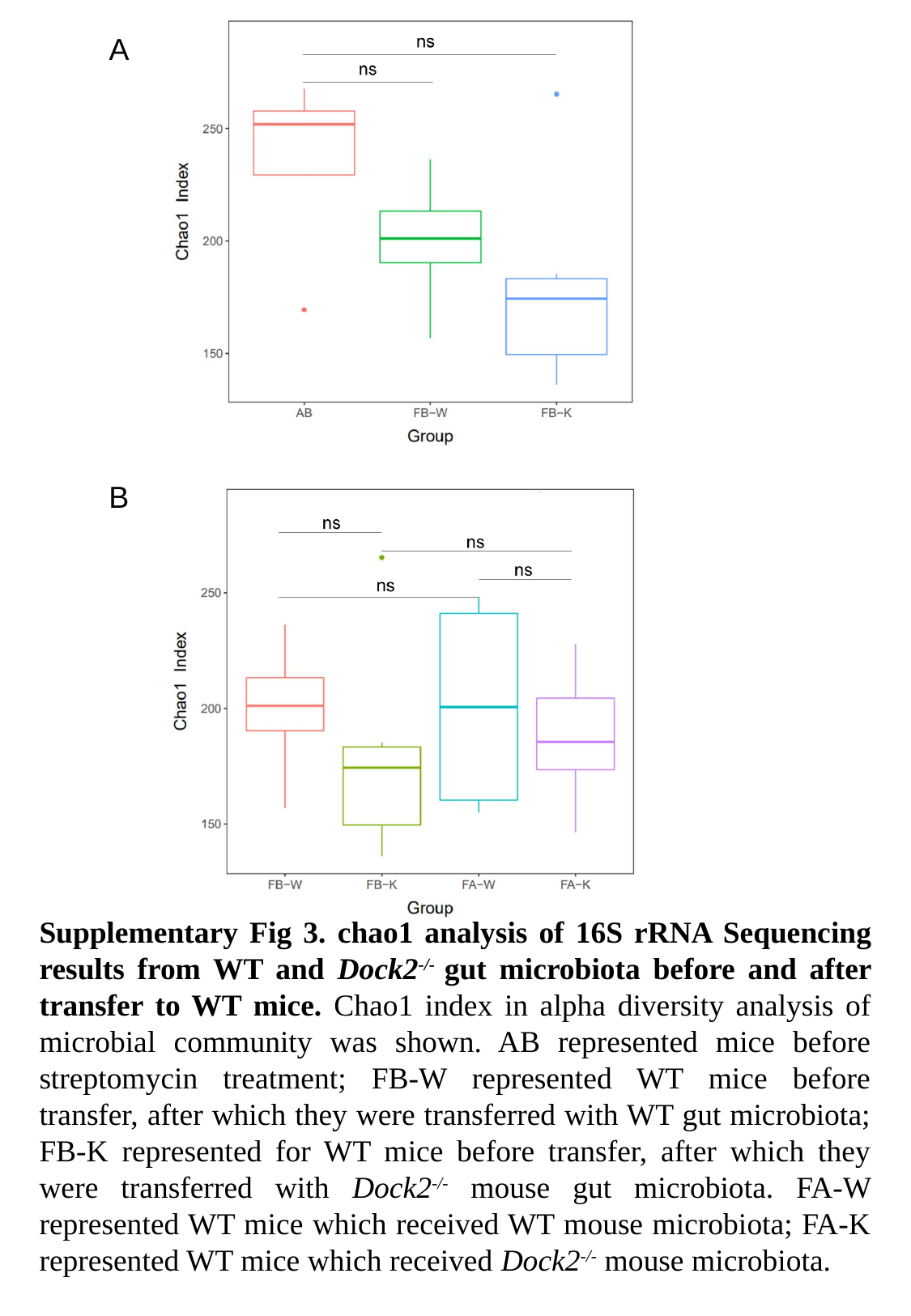

A
B
Supplementary Fig 3. chao1 analysis of 16S rRNA Sequencing results from WT and Dock2-/- gut microbiota before and after transfer to WT mice. Chao1 index in alpha diversity analysis of microbial community was shown. AB represented mice before streptomycin treatment; FB-W represented WT mice before transfer, after which they were transferred with WT gut microbiota; FB-K represented for WT mice before transfer, after which they were transferred with Dock2-/- mouse gut microbiota. FA-W represented WT mice which received WT mouse microbiota; FA-K represented WT mice which received Dock2-/- mouse microbiota.
